# Knowledge, attitudes, and practices regarding mosquito-borne diseases in an urban sector of southwestern Colombia

**DOI:** 10.3389/fpubh.2025.1682827

**Published:** 2025-12-03

**Authors:** Francisco Javier Bedoya-Rodríguez, Carlos Eduardo Guevara-Fletcher, Jonathan S. Pelegrin

**Affiliations:** 1 Grupo de Investigación en Ecología y Conservación de la Biodiversidad (EcoBio), Equipo de Paleobiología, Ecología y Evolución (PaleoEco), Facultades de Ciencias Básicas y Educación, Universidad Santiago de Cali, Campus Pampalinda, Cali, Colombia; 2 Escuela de Ingeniería de Sistemas y Computación, Facultad de Ingeniería, Universidad del Valle, Sede Norte, Santander de Quilichao, Colombia; 3 Escuela de Ciencias Agrícola Pecuarias y del Medio Ambiente, Universidad Nacional Abierta y a Distancia, Cali, Colombia

**Keywords:** arboviruses, health and biological education, mosquito control, knowledge, attitudes and practices, mosquito-borne diseases

## Abstract

**Background:**

Mosquito-borne diseases (MBD) continue to represent a critical public health challenge, particularly in tropical regions where environmental and socioeconomic factors facilitate transmission.

**Methods and materials:**

This study assessed the knowledge, attitudes, and practices (KAP) related to MBD among 172 residents of an urban area in Santander de Quilichao, Colombia. A structured survey, validated and adapted from the Pan American Health Organization, was performed for data collection.

**Results:**

The mean knowledge score was 57%, indicating moderate awareness of MBD. While 63.4% of participants reported adequate preventive practices, the majority (94.8%) demonstrated indifferent attitudes toward MBD prevention. Multinomial logistic regression analysis revealed a statistically significant association between marital status and knowledge levels (*p* < 0.05), with married participants exhibiting higher knowledge. Socioeconomic analysis showed that 41.3% of participants earned the legal minimum wage, and 68.6% of households included at least one woman of childbearing age. Despite moderate knowledge and preventive practices, the prevalence of indifferent attitudes may hinder effective MBD prevention.

**Conclusion:**

These findings emphasize the need for targeted community engagement and educational interventions. Incorporating KAP assessments into public health strategies can enhance the design and implementation of effective educational and vector control programs in urban areas of tropical countries.

## 1 Introduction

Globally, arthropod-borne vector-borne diseases (VBD) represent a public health challenge, with an estimated 700,000 deaths per year. Approximately 17% of the world’s infectious diseases are attributable to VBD (1). Approximately 80% of the global population resides in areas with a high risk of VBD transmission, particularly in tropical and subtropical regions (1, 2). The persistent threat of these diseases, particularly mosquito-borne diseases (MBD), is exacerbated by factors such as inadequate public awareness and the spread of misinformation, which hinder effective prevention and control efforts (1). Mosquitoes (Diptera, Culicidae) are the primary vectors for numerous MBD, including dengue, Zika, chikungunya, and yellow fever (transmitted by *Aedes* spp.), as well as malaria (*Anopheles* spp.), Japanese encephalitis, and West Nile virus (both mainly transmitted by Culex spp.) (1, 3–5). These arboviruses, a diverse group encompassing the *Peribunyaviridae*, *Flaviviridae*, *Togaviridae*, *Reoviridae*, and *Orthomyxoviridae* families (6), represent a significant challenge to healthcare systems worldwide. The increasing overlap in the geographical distribution of these diseases creates diagnostic challenges and can complicate clinical management (7).

In Americas, the most prevalent arboviruses are the dengue, Zika, and chikungunya viruses (8). These viruses are primarily transmitted by *Aedes* mosquitoes, especially *Aedes aegypti* (Linnaeus, 1762) and *Aedes albopictus* (Skuse, 1894), and have spread extensively throughout the region (9). While dengue virus is the most widespread, with an estimated 96 million cases annually, chikungunya and Zika also contribute significantly to the disease burden (10). The Americas and Caribbean reported in 2024, 13,027,747 cases, approximately three times higher than the previous year (11). Also, 16,239 cases of Oropouche (102) and 186,274 cases of chikungunya were reported at the same year (12). The rapid increase in the cases, with the presence of other arboviruses, such as yellow fever virus, West Nile virus, and those causing equine encephalitis (8), showing the urgent need for improved surveillance and control strategies. The rapid distribution of *Ae. aegypti* and *Ae. albopictus* has amplified the arboviral diseases (9). Other aspects, such as climate change, and urbanization increase in natural areas present a challenge to disease control and prevention (13). Notwithstanding the implementation of environmental controls and educational strategies (14), community participation in activities of adoption of preventive practices are imperative (15, 16).

Studies on knowledge, attitudes, and practices (KAP) related to MBD have been important tools for developing public health interventions both urban and rural areas (17). Various studies have shown the important of the educational process in preventing and mitigating of MBD across diverse socio-environmental and cultural contexts (18–22). KAP studies are frequently used to assess community knowledge, behaviors, and responses to MBD (23). Van den Berg et al. (24) carried out studies on insecticide management practices to control mosquito. They founded poor knowledge about its use demonstrating the necessity to involve the community in good management to eradicate mosquito.

The significance of KAP studies is widely acknowledged due to their relationship with social and cultural factors that influence the understanding of environmental challenges (25). In Asia, KAP studies realized in Bangladesh have evaluated public perspectives on dengue dynamics across different educational and industrial sectors (26). These studies have demonstrated that KAP can significantly enhance knowledge, promote preventive actions, and refine public health strategies (27–29). Furthermore, KAP studies have been related with socioeconomic factors and climate change impacts both urban and rural areas (30–35). A comparative study involving non-endemic (Turkey) and dengue-endemic countries (Bangladesh, India, and Malaysia) demonstrated the value of KAP in improving dengue diagnosis and treatment within healthcare systems (36). Analogous KAP analyses on dengue have been conducted in other endemic regions, including Yemen and Malaysia, as well as non-endemic regions, such as Hong Kong (37–39). Other studies carried out in China, Vietnam, India, and Singapore have investigated beliefs, behaviors, perceptions, willingness, and awareness related to MBD prevention and control (40–44). The collection of KAP data demonstrated that the development of educative strategies enhancing knowledge and promoting behavioral change between human and mosquitoes (45). This assertion is confirmed by studies conducted in diverse settings, including patient populations, healthcare facilities, and educational institutions (46–48).

In Africa, some studies affirmed that scarce ecological knowledge regarding MBD carry to use pesticide and not to implement educational strategies (28). In Tanzania, studies were focus on KAP with the purpose of prevent dengue and to understand mosquito ecology (49). In Kenya, the studies have linked knowledge, beliefs and management of MBD to socioeconomic factors within the livestock sector (50). In the Americas, KAP studies carried out in countries such as United States, Mexico, Brazil, and Peru have examined preventive behaviors in relation to beliefs, personality traits, social responsibility, and sociodemographic factors across urban, rural, and marginalized contexts (51–55). In Colombian where MBD are endemic, KAP studies have related knowledge with sociodemographic characteristics, risk perception, and population experiences (56–60).

In order to better understand and address the impact of mosquitoes on human health, it is essential to analyze MBD using the KAP framework. Assessing KAP can inform strategies aimed at preventing and controlling mosquito populations by promoting positive changes in human behavior (61). This research purpose to analyze the role of neighborhood and individual factors in the KAP related to MDB in the municipality of Santander de Quilichao, Cauca, southwest Colombia, using community surveys. The climate in this area is favorable for the presence of MBD, particularly dengue and malaria (62, 63).

## 2 Materials and methods

### 2.1 Study area and participants

**Figure 1:**
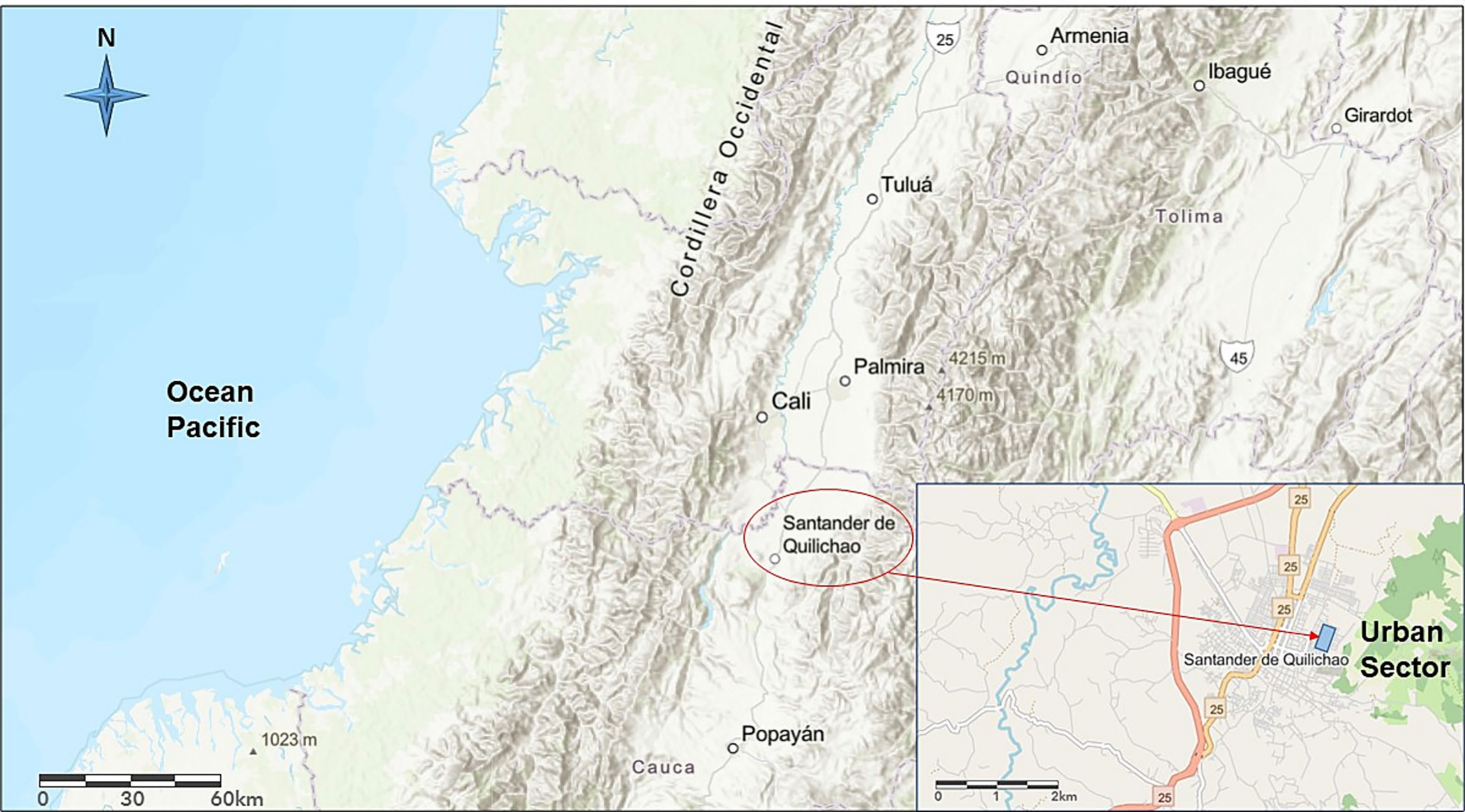
Location of the study area in Santander de Quilichao, Department of Cauca, southwestern Colombia. The study area is identified (blue rectangle).

The KAP community survey was conducted in two neighborhoods in the urban area of Santander de Quilichao, located in the Cauca department, in southwestern Colombia (3°00′32.0”N 76°28′39.4”W) (Figure 1). The neighborhoods were selected due to its socioeconomic status (low middle income), which represents a significant segment of the city’s population (62). The study area is situated in tropical deciduous woodland bio-climatic zone. The survey was conducted from October 2022 to February 2023. This municipality has a total population of 53,856 inhabitants in the urban area (63).

The sample size was calculated using the formula for estimating a proportion in a finite population, with a 95% confidence level (*Z* = 1.96), and a margin of error of 5%. For the 310 households in the study area, this yielded a required sample size of 172. The sample size was verified using the online SurveyMonkey application.^1^ Households were selected by simple random sampling from the municipal census list. Each household was contacted by telephone or visited directly if no phone number was available. Eligible participants were adults (≥18 years), responsible for the household, and residents of the community with access to a mobile phone and internet. If a household declined or could not be reached, another was randomly selected to ensure the sample size was achieved. The main hypothesis was that sociodemographic factors influence KAP regarding MBD.

### 2.2 Questionnaire design

The survey used in this study was designed using Google Forms.^2^ The design process included elements such as informed consent, socio-demographic data and a series of questions on KAP related to MBD and mosquito control (Supplementary Table 1). The survey instrument used in this study was adapted from the KAP questionnaire developed by the Pan American Health Organization (64). This instrument has been previously validated and widely employed in mosquito-borne disease research, providing a robust framework for assessing community knowledge and behaviors related to vector control. The survey provided a foundation upon which the questionnaire was adapted through a series of expert consultations with local entomologists, public health officials, and community leaders. The objective of these survey was to show the relevance of the questionnaire at the local context and the specific MBD prevalent in the region (64). A pilot study was conducted with 30 households according to Bujang et al. (65), in order to know the reliability, clarity, and cultural appropriateness of the questionnaire. The feedback from the pilot study was used to refine the questionnaire before the main data survey. Specifically, we modified items related to disease symptomatology to better reflect locally prevalent illnesses, simplified wording in attitude-related questions to enhance comprehension, and added culturally relevant examples within practice items. These adjustments aimed to optimize the instrument’s clarity, relevance, and respondent engagement. Cronbach’s alpha was calculated to analyze the internal consistency of knowledge, attitude, and practice scales (acceptable at 70.2%). Exploratory factor analysis (EFA) was performed with Varimax rotation to determine construct validity (23). These analyses will provide evidence of the questionnaire’s reliability and validity.

The research met ethical standards with approval from the Ethics Committee of the Faculty of Education at the University of Santiago de Cali and the local Community Action Committee. Informed consent was obtained via Google Forms, and an unbiased sample was maintained by randomly replacing non-participating households.

The questionnaire included both general items on mosquito-borne diseases and disease-specific items, particularly related to dengue. This approach was chosen due to the high prevalence of dengue in the study area at the time of data collection. The questionnaire was divided in four sections: sociodemographic characteristics (nine questions), knowledge (12 questions), attitudes (11 questions), and practices (eight questions) respect to MBD and mosquito control. The sociodemographic section included variables such as gender, age, marital status, education level, occupation, number of dependents, monthly income, and the number of reproductive women, and pregnant women. These data allow understand how these factors may influence KAP regarding MBD. A knowledge index was developed based on 10 most relevant questions, with ranging between 0 and 10 (44). Each correct or positive answer received a score of 1, while incorrect or negative answer received a score of 0. This scoring system facilitated the categorization of knowledge into six sub-categories as follows: 0 (without knowledge), 1–2 (very low knowledge), 3–4 (low knowledge), 5 (medium knowledge), 6–8 (high knowledge), and 9–10 (very high knowledge). Questions 11 and 18 were designed to assess participants’ comprehension of mosquito biology and their disease transmission. Questions 17 and 19 analyzed the dengue and its prevalence in the study area. In this context, responses were organized such as ‘correct and indifferent’ (1) or ‘incorrect’ (0), thereby contributing to the calculation of the overall knowledge score. In this case, ‘indifferent’ responses indicate partial knowledge without misinformation, and were rated as correct (1) in the knowledge index to distinguish uncertainty from incorrect knowledge. Question 10 asked participants if they were familiar with the term mosquito-borne disease (MBD). Those who responded negatively were excluded from the knowledge score analysis, as their lack of basic awareness rendered subsequent knowledge items inapplicable, ensuring the accuracy and validity of the knowledge assessment. This methodological approach was taken to ensure an accurate assessment of knowledge levels and to maintain the integrity of the study.

The classification of attitudes was divided into three subcategories. The scores from 0 to 14 indicated a negative attitude, scores from 15 to 34 indicated an indifferent attitude, and scores from 35 to 44 reflected a positive attitude toward MBD and mosquito control. The responses to the Practice Index questions were categorized and scored as follows: each affirmative or correct practice was assigned one point, resulting in a cumulative score ranging from 0 to 8. Scores ranging from 0 to 2 indicated inadequate practices, scores from 3 to 5 reflected moderate practices, and scores from 6 to 8 indicated adequate practices. The thresholds for attitude and practice scores were determined using a combination of adaptations from previous CAP studies (19, 30, 34, 44), ensuring their contextual relevance and replicability.

### 2.3 Data analysis

In order to analyze the relationship between sociodemographic variables and knowledge levels, we employed a multinomial logistic regression model. The knowledge index, categorized into five levels, served as the dependent variable, with the lowest category used as the reference. All independent variables were included simultaneously in the model, including gender, age, marital status, education, occupation, income, number of dependents, presence of reproductive women, and pregnant women in the household. This approach enables the estimation of the effect of each predictor on the odds of belonging to each knowledge category relative to the reference. The same set of variables was applied across all outcome categories, ensuring the comparability and interpretability of the associations. The regression model was built using gender, age, marital status, education and occupation as independent variables. These factors have been consistently identified in the literature as the most robust and theoretically relevant predictors of knowledge, attitudes and practices regarding MBD (c, 39, 51). Other registered variables were not included in the model because they did not demonstrate sufficient variability or statistical association in preliminary analyses.

A comprehensive statistical analysis was conducted to explore the statistical association between socio-demographic factors and KAP outcomes. Thus, descriptive statistical analyses were used to describe the relationships between different socio-demographic aspects and KAP scores. Prior to conducting the multinomial logistic regression, bivariate analyses were performed to examine the relationship between each socio-demographic variable and the levels of KAP. Chi-square tests were used to assess the association between categorical variables (marital status, gender, occupation, education level) and KAP levels. Variables with a *p*-value < 0.05 in the bivariate analyses were selected for inclusion in the multinomial logistic regression model. Variance inflation factors (VIF) were calculated to assess multicollinearity among the independent variables, if VIF values exceeded 5, the variable with the highest VIF was considered for removal from the model. Specifically, a multinomial logistic regression model was used, implemented through the SPSS version 25 statistical package. In this model, socio-demographic variables served as independent variables, while KAP scores served as dependent variables. This analytical approach aimed to uncover significant associations between these variables, thereby providing insights into how socio-demographic characteristics influence KAP levels within the studied population (66).

## 3 Results

### 3.1 Instrument reliability and validation on KAP dimensions

To ensure the questionnaire’s relevance and cultural appropriateness, consultations were conducted with local entomologists, public health officials, and community leaders. The consensus and agreement from these consultations were quantitatively evaluated using Cohen’s Kappa coefficient for inter-rater reliability, yielding a substantial agreement with a Kappa value of 0.78 (95% CI: 0.70–0.85), which indicated high concordance among experts. Feedback from these consultations guided the refinement of specific questionnaire items to improve clarity and local contextual relevance.

Cronbach’s alpha coefficients for the knowledge, attitude, and practice scales were 0.70, 0.75, and 0.79, respectively, indicating acceptable to good internal consistency. An exploratory factor analysis (EFA) was performed on the CAP scale items to evaluate their underlying factor structure and the construct validity of the instrument. A Varimax rotation analysis was applied in order to identify the three factor dimensions that constitute the instrument. The analysis yielded a three-factor solution that explained 86.27% of the total variance. Please refer to Table 1, which presents the factor loadings grouped according to the CAP dimensions. The knowledge dimension grouped 12 items and explained 46.55% of the variance, with average factor loadings around 0.53, indicating an adequate association between the items and that dimension. The attitude dimension, which comprises 11 items, contributed 16.51% of the variance and had an average factor loading close to 0.75, reflecting a strong relationship between the items and this factor. The practice dimension, consisting of eight items, explained 23.22% of the total variance and showed the highest average factor loadings (0.79), evidencing a strong link between preventive measures and this dimension. All items exhibited clear and interpretable factor loadings, consistent with their theoretical assignment. Following the analysis, no items were excluded or modified, indicating stable psychometric properties and robust construct validity.

**Table 1:** Summary of average factor loadings and variance explained by dimension in exploratory factor analysis (Varimax rotation).

Dimension	Number of items	Explained variance (%)	Approximate average factor loadings
Knowledge	12	46.55	0.53
Attitude	11	16.51	0.75
Practice	8	23.22	0.79

### 3.2 Sociodemographic characteristics and KAP

Key variables such as age, gender distribution, and educational level are summarized to provide a concise overview, providing the participants’ demographics and offering essential context for further analysis (Table 2). In Colombia, “technical” education is short-term and skills-focused, while “technological” education is more advanced and prepares students for mid-level professional roles.

**Table 2:** Sociodemographic characterization of the target population in a local area from municipality of Santander de Quilichao, Colombia.

Category	Description	Frequency	Percentage (%)
Gender	Men	74	57.0
	Women	98	43.0
Age range	18 to 27 years old	34	19.8
	28 to 37 years old	56	32.6
	38 to 47 years old	56	32.6
	Over 48 years old	26	15.0
Marital status	Unmarried	65	37.8
	Married	56	32.5
	Free union	43	25.0
	Separated	6	3.5
	Widowed	2	1.2
Level of education	University	102	59.3
	Technological	19	11.1
	Technical	30	17.4
	Secondary	14	8.1
	Primary	1	0.6
	Other	6	3.5
Occupation	Employees	98	57.0
	Self-employed	50	29.1
	Various trades	8	4.6
	Other	16	9.3
Average monthly income	Legal economic income (LEI) (U$340 approx.)	71	41.3
	Between 1 and 2 LEI	53	30.8
	Between 3 and 4 LEI	44	25.6
	None	4	2.3
Reproductive women (15 to 49 years old)	Households with at least one reproductive woman	118	68.6
	Households with a pregnant woman	3	1.7
Mobile phones	Population with a mobile phone		100.0

### 3.3 Knowledge of mosquito-borne diseases

A total of 62.8% of participants, were aware of MBD. Moreover, 91.9% are aware of the life cycle of mosquitoes, which are critical vectors in both natural and man-made environments. While 57.6% of participants acquired knowledge of those cycles many years ago, only 12.8% learned about them during the surveys. The information about MBD was obtained mainly by TV (31.4%), followed by interpersonal (24.4%) and family (19.2%) communications. Social networks and internet represented 18.6 and 5.4%, respectively. Perceived susceptibility to MBD was reported by 66.9% of participants; in contrast, 21.5% were ambivalent and 9.9% were unaware of their risk status. Furthermore, when asked about personal experience within their community, 66.9% did not know anyone who had sickness by MBD.

Interestingly, while 91.9% acknowledge that anyone can contract MBD, only 21.5% correctly identify dengue as a viral disease, despite its strong association with mosquito transmission (90.1%). Knowledge of dengue symptoms such as fever and muscle pain were recognized by the population (60%); however, the 12.2% remain uninformed about these symptoms. Preventive strategies against MBD are mainly focused on environmental management, with 84.9% supporting the elimination of stagnant water as the most important strategy, followed by cleaning water storage containers (75.6%) and fumigation efforts (71.5%). According to 58.7% of respondents, treatment for MBD is available. With respect to knowledge of MBD, the participants showed very high (9.9%), high (57%) medium (15%) and between low and very low (16.3%) levels.

### 3.4 Attitudes on mosquito-borne diseases

The majority of interviewees (58.5%) responded that MBD are an important problem for the community, 29.1% was agreed, 9.9% were indifferent, 1.2% disagreed and 4.1% totally disagreed. The probability of contracting a MBD in the next six-month, were as follows: 21.1% expressed a strong agreement, 23.8% indicated an agreement, 34.3% remained indifferent, 14.5% expressed disagreement, and 5.2% strongly disagreed. In the moment to contract a MBD, 13.4% were strongly agreed that the information should remain confidential, 13.4% agreed, 29.1% were indifferent, 18.6% disagreed, and 25.6% strongly disagreed. In cases of family could be discriminated for contracting MBD, they responded that 72.1% strongly disagreed, 12.2% disagreed, and 8.1% were indifferent. 90% considered that the responsibility of each individual to adopt preventive measures to avoid MBD, followed by health staff (24.4%). At home, the responsibility is personal (62.2%), or head of household (46.5%) and healthcare providers (16.3%). The responsibility in the community is to, municipal administration (43.6%), personal responsibility (41.3%), health personnel (35.5%) and community leaders (33.7%). The question about the effective treatment of individuals infected with MBD, showed that the treatment should be administered in a hospital (72.7%), by a private doctor (49.4%), in a public health center (36%), in a pharmacy (8.1%) or by an unqualified practitioner (1.7%). It was evident that 51.7% of the participants did not have enough information about MBD. The 94.8% displayed an indifferent attitude, while 5.2% showed a positive attitude (Table 3).

**Table 3:** Summary of key attitudes toward MBD.

Evaluated attitude	Response	Percentage (%)
Importance of MBD for the community	Important	58.5
	Agree	29.1
	Indifferent	9.9
	Disagree	1.2
	Strongly disagree	4.1
Probability of contracting MBD in 6 months	Strongly agree	21.1
	Agree	23.8
	Indifferent	34.3
	Disagree	14.5
	Strongly disagree	5.2
Confidentiality of information when contracting MBD	Strongly agree	13.4
	Agree	13.4
	Indifferent	29.1
	Disagree	18.6
	Strongly disagree	25.6
Family discrimination for contracting MBD	Strongly disagree	72.1
	Disagree	12.2
	Indifferent	8.1
Responsibility for adopting preventive measures	Individual	90
	Health staff	24.4
Responsibility at home	Individual	62.2
	Head of household	46.5
	Healthcare providers	16.3
Responsibility in the community	Municipal administration	43.6
	Individual	41.3
	Health personnel	35.5
	Community leaders	33.7
Place where treatment should be administered	Hospital	72.7
	Private doctor	49.4
	Public health center	36
	Pharmacy	8.1
	Unqualified practitioner	1.7
Sufficient information about MBD	Does not have enough	51.7
Attitude toward MBD	Indifferent	94.8
	Positive	5.2

### 3.5 Practices on mosquito-borne diseases

A total of 72.1% of participants carried out preventive measure to avoid contracting MBD. The main methods of protection were (a) Elimination of stagnant water: 94.2% considered that eliminating all types of stagnant water reduce the mosquito populations, (b) Personal protection: 88.4% protecting themselves from MBD. 10.5% of participants considered that local organizations were doing enough to prevent MBD, represented by local government (11.6%) and national government (13.4%). 75.6% reported cleaning tanks, storage containers or stagnant water the last week. This behavior is crucial in reducing potential breeding sites for mosquito and reflects a commitment to maintaining a healthier environment. The survey showed a strong willingness among participants to participate in vaccination campaigns against MBD, 76.7% accepting a vaccine if is available. Adequate practices for the prevention of MBD were observed in 63.4% of participants, whereas 36.6% had moderate practices (Table 4).

**Table 4:** Summary of key practices in MBD prevention.

Evaluated practice	Response	Percentage (%)
Perform preventive measures to avoid MBD	Yes	72.1
Main methods of protection	Elimination of stagnant water	94.2
	Personal protection	88.4
Perception of local organizations in prevention	Considered to be doing enough	10.5
	Local government	11.6
	National government	13.4
Cleaning tanks or containers last week	Performed	75.6
Willingness to participate in vaccination campaigns	Accept vaccine if available	76.7
Observed adequacy of prevention practices	Adequate practices	63.4
	Moderate practices	36.6

### 3.6 Association between sociodemographic variables and KAP

There were differences in knowledge levels about MBD according to key sociodemographic variables, specifically age (38 to 47 years old) and marital status. The results demonstrated a highly significant relationship between higher knowledge levels and being aged between 38 and 47 years (*χ*^2^ = 446.211, *p* < 0.001). Similarly, marital status showed significant associations: unmarried (*χ*^2^ = 93.193, *p* < 0.001), married (*χ*^2^ = 100.373, *p* < 0.001), and living in a free union (*χ*^2^ = 96.674, *p* < 0.001). Additionally, no significant relationship was found between any of the sociodemographic variables and either attitude or practice toward MBD.

Bivariate analyses revealed statistically significant relationship between marital status (*p* < 0.001) and age (*p* = 0.02) with knowledge levels. Multinomial logistic regression analysis (Table 5) showed that marital status is a critical factor influencing knowledge about diseases, particularly among participants with high knowledge. For example, individuals aged 38 to 47 years had markedly higher odds of high knowledge compared to those over 48 years [Exp(B) = 6.43 × 10^7^; 95% CI: 9.27 × 10^6^–4.45 × 10^8^; *p* < 0.001]. Conversely, being unmarried substantially decreased the odds of high knowledge [Exp(B) = 4.68 × 10^−13^; 95% CI: 4.48 × 10^−15^–4.89 × 10^−11^; *p* < 0.001]. Other factors, such as gender and occupation, were not significant predictors across all knowledge levels. Although education level showed positive trends, significance was only marginal for low and very high knowledge groups (*p* = 0.068 and 0.075, respectively).

**Table 5:** Multinomial logical regression analysis of sociodemographic factors associated with knowledge about MBD in a local area from Santander de Quilichao, Colombia.

Dependent variable	Independent variable	Coefficient B	Standard error	Exp(B) (adj. OR)	95% CI lower	95% CI upper	Statistician Wald	*p*-value
High knowledge	Gender (man)	0.372	1.429	1.451	0.056	37.495	0.068	0.795
	Gender (woman) (ref)	0b	–	–	–	–	–	–
	Age (18 to 27 years old)	1.473	4.003	4.362	0	3081.15	0.135	0.713
	Age (28 to 37 years old)	−0.973	2.703	0.378	0.006	21.64	0.13	0.719
	Age (38 to 47 years old)	**17.996**	**0.852**	**6.43E+07**	**9.27E+06**	**4.45E+08**	**4.46E+02**	**<0.001**
	Age (over 48 years old)	0b	–	–	–	–	–	–
	Marital status (unmarried)	**−28.385**	**2.94**	**4.68E-13**	**4.48E-15**	**4.89E-11**	**9.32E+01**	**<0.001**
	Marital status (married)	**−28.831**	**2.878**	**3.12E-13**	**3.62E-15**	**2.68E-11**	**1.00E+02**	**<0.001**
	Marital status (free union)	**−28.68**	**2.917**	**3.55E-13**	**3.64E-15**	**3.46E-11**	**9.67E+01**	**<0.001**
	Marital status (separated)	−1.034	8.594	0.356	4.70E-08	2691.3	0.014	0.904
	Marital status (widowed)	0b	–	–	–	–	–	–
	Level of education (primary school)	0.909	0.607	2.482	0.732	8.41	2.238	0.135
	Level of education (secondary)	−0.473	80.692	0.623	0	Huge CI	0	0.995
	Level of education (technician)	0.793	3.278	2.21	0	Huge CI	0.059	0.809
	Level of education (technologist)	3.449	4.182	31.5	0	Huge CI	0.68	0.41
	Level of education (university)	−2.55	4.718	0.078	0	Huge CI	0.292	0.589
	Level of education (other)	0b	–	–	–	–	–	–
	Occupation (employed)	−0.441	0.652	0.644	0.193	2.148	0.459	0.498
	Occupation (self-employed)	0.333	5.25	1.395	0	Huge CI	0.004	0.949
	Occupation (various trades)	1.321	5.375	3.747	0	Huge CI	0.06	0.806
	Occupation (other)	0b	–	–	–	–	–	–
Medium knowledge	Gender (man)	0.693	1.472	2	0.091	43.815	0.222	0.638
	Gender (woman) (ref)	0b	–	–	–	–	–	–
	Age (18 to 27 years old)	2.223	4.085	9.235	0	Huge CI	0.296	0.586
	Age (28 to 37 years old)	−0.341	2.826	0.711	0.016	32.088	0.015	0.904
	Age (38 to 47 years old)	**18.83**	**1.126**	**1.53E+08**	**2.10E+07**	**1.11E+09**	**2.80E+02**	**<0.001**
	Age (over 48 years old)	0b	–	–	–	–	–	–
	Marital status (unmarried)	0.462	1.029	1.587	0.207	12.13	0.201	0.654
	Marital status (married)	0.765	14.755	2.149	0	Huge CI	0.003	0.959
	Marital status (free union)	0.358	14.741	1.431	0	Huge CI	0.001	0.981
	Marital status (separated)	2.095	14.928	8.141	0	Huge CI	0.02	0.888
	Marital status (widowed)	0b	–	–	–	–	–	–
	Level of education (primary school)	0.63	0.619	1.877	0.533	6.594	1.037	0.309
	Level of education (secondary)	7.992	80.884	2964.59	0	Huge CI	0.01	0.921
	Level of education (technician)	0.296	4.123	1.344	0	Huge CI	0.005	0.943
	Level of education (technologist)	4.642	4.184	103.95	0.007	1.44E+06	1.23E+00	0.267
	Level of education (university)	1.575	2.186	4.833	0.283	82.55	0.519	0.471
	Level of education (other)	0b	–	–	–	–	–	–
	Occupation (employed)	0	0.663	1	0.302	3.312	0	1
	Occupation (self-employed)	−0.231	5.282	0.794	0	Huge CI	0.002	0.965
	Occupation (various trades)	1.308	5.399	3.698	0	Huge CI	0.059	0.809
	Occupation (other)	0b	–	–	–	–	–	–
Low knowledge	Gender (man)	1.499	17.438	4.48	0	Huge CI	0.007	0.931
	Gender (woman) (ref)	0b	–	–	–	–	–	–
	Age (18 to 27 years old)	1.734	4.114	5.66	0	Huge CI	0.178	0.673
	Age (28 to 37 years old)	−0.739	2.825	0.478	0.031	7.327	0.068	0.794
	Age (38 to 47 years old)	18.36	1.159	9.50E+07	1.27E+07	7.13E+08	2.51E+02	<0.001
	Age (over 48 years old)	0b	–	–	–	–	–	–
	Marital status (married)	−0.596	16.664	0.551	0	Huge CI	0.001	0.971
	Marital status (free union)	−0.884	16.659	0.413	0	Huge CI	0.003	0.958
	Marital status (separated)	0.155	16.83	1.168	0	Huge CI	0	0.993
	Marital status (widowed)	−2.502	27.452	0.081	0	Huge CI	0.008	0.927
	Marital status (unmarried)	0b	–	–	–	–	–	–
	Level of education (primary school)	1.187	0.651	3.28	0.938	11.48	3.325	0.068
	Level of education (secondary)	−1.598	80.685	0.202	0	Huge CI	0	0.984
	Level of education (technician)	0.53	2.993	1.699	0	Huge CI	0.031	0.859
	Level of education (technologist)	2.278	4.196	9.757	0	Huge CI	0.295	0.587
	Level of education (university)	−3.303	4.535	0.037	0	Huge CI	0.53	0.466
	Level of education (other)	0b	–	–	–	–	–	–
	Occupation (employed)	−0.042	0.672	0.959	0.264	3.485	0.004	0.951
	Occupation (self-employed)	−0.896	5.302	0.408	0	Huge CI	0.029	0.866
	Occupation (various trades)	0.428	5.422	1.534	0	Huge CI	0.006	0.937
	Occupation (Other)	0b	–	–	–	–	–	–

Significant factors are shown in bold. b, reference category for each independent variable.

The results from Tables 6, 7 indicate that there were no statistically significant associations between sociodemographic variables and either attitudes or practices regarding MBD in the studied population. Specifically, none of the independent variables-including gender, age, marital status, level of education, or occupation-showed significant effects on having a positive attitude (Table 6) or adequate practice (Table 7), consistent with high *p*-values and Exp(B) values near unity.

**Table 6:** Multinomial logical regression analysis of sociodemographic factors associated with attitude about MBD in a local area from Santander de Quilichao, Colombia.

Dependent variable	Independent variable	Coefficient B	Standard error	Exp(B) (adj. OR)	95% CI lower	95% CI upper	Statistician Wald	*p*-value
Positive attitude	Gender (man)	−0.537	0.784	0.585	0.161	2.129	0.469	0.49
	Gender (woman)	0b	–	–	–	–	–	–
	Age (18 to 27)	0.05	1.347	1.051	0.107	10.34	0.001	0.97
	Age (28 to 37)	0.067	1.277	1.069	0.099	11.59	0.003	0.96
	Age (38 to 47) (neg)	−0.835	1.563	0.434	0.054	3.467	0.286	0.59
	Age (over 48)	0b	–	–	–	–	–	–
	Marital status (unmarried)	16.541	7231.451	Very large*	Very large*	Very large*	0	1
	Marital status (married)	0.153	7392.654	1.165	Very large*	Very large*	0	1
	Marital status (free union)	16.475	7231.451	Very large*	Very large*	Very large*	0	1
	Marital status (separated)	−0.473	8861.947	0.623	Very large*	Very large*	0	1
	Marital status (widowed)	0b	–	–	–	–	–	–
	Level of education (primary school)	12.483	4051.594	Very large*	Very large*	Very large*	0	1
	Level of education (secondary)	15.667	4051.594	Very large*	Very large*	Very large*	0	1
	Level of education (technician)	14.91	4051.594	Very large*	Very large*	Very large*	0	1
	Level of education (technologist)	−0.238	4817.605	0.788	0	Very large*	0	1
	Level of education (university)	15.793	4051.594	Very large*	Very large*	Very large*	0	1
	Level of education (other)	0b	–	–	–	–	–	–
	Occupation (employed)	0.659	1.336	1.933	0.225	16.56	0.243	0.62
	Occupation (self-employed)	−0.417	1.412	0.659	0.097	4.482	0.087	0.77
	Occupation (various trades)	−15.166	3759.75	Very small*	Very small*	Very small*	0	1
	Occupation (other)	0b	–	–	–	–	–	–

The reference category is indifferent attitude. b, reference category for each independent variable.

**Table 7:** Multinomial logical regression analysis of sociodemographic factors associated with practice about MBD in a local area from Santander de Quilichao, Colombia.

Dependent variable	Independent variable	Coefficient B	Standard error	Exp(B) (adj. OR)	95% CI lower	95% CI upper	Statistician Wald	*p*-value
Adequate practice	Gender (man)	−0.551	0.377	0.576	0.264	1.259	0.977	0.446
	Gender (woman)	0b	–	–	–	–	–	–
	Age (18 to 27)	−0.59	0.737	0.554	0.164	1.873	0.64	0.424
	Age (38 to 47)	−0.828	0.657	0.437	0.122	1.565	1.588	0.208
	Age (38 to 47) (neg)	−1.018	0.637	0.361	0.133	0.978	2.556	0.11
	Age (over 48)	0b	–	–	–	–	–	–
	Marital status (unmarried)	0.787	1.495	2.198	0.127	38.08	0.277	0.599
	Marital status (married)	1.218	1.511	3.379	0.172	66.36	0.65	0.42
	Marital status (free union)	1.489	1.53	4.433	0.201	97.69	0.947	0.331
	Marital status (separated)	19.496	6234.51	Very large*	Very large*	Very large*	0	0.998
	Marital status (widowed)	0b	–	–	–	–	–	–
	Level of education (primary school)	0.828	1.6	2.289	0.114	45.78	0.325	0.458
	Level of education (secondary)	0.196	1.208	1.217	0.129	11.56	0.026	0.871
	Level of education (technician)	−0.533	1.022	0.587	0.071	4.851	0.272	0.602
	Level of education (technologist)	0.081	1.059	1.084	0.112	10.49	0.006	0.939
	Level of education (university)	−0.218	0.937	0.804	0.207	3.117	0.054	0.816
	Level of education (other)	0b	–	–	–	–	–	–
	Occupation (employed)	0.048	0.718	1.049	0.295	3.727	0.005	0.946
	Occupation (self-employed)	0.355	0.71	1.426	0.377	5.381	0.249	0.617
	Occupation (various trades)	0.351	0.312	1.421	0.774	2.608	0.518	0.511
	Occupation (other)	0b	–	–	–	–	–	–

The reference category is moderate practice. b, reference category for each independent variable.

## 4 Discussion

Sociodemographic factors significantly influence KAP regarding MBD, informing effective interventions in tropical and subtropical regions (31). Community participation in various activities related to the prevention and mitigation of MBD is crucial for maintaining public health (77). A significant proportion of participants in this study identified mosquitoes as the primary vector of MBD (18, 31, 103, 104). In addition, knowledge of dengue symptoms was widespread, consistent with the findings of Selvarajoo et al. (38). Television, followed by social media and the Internet, were important sources of information about MBD and mosquito control. This is consistent with studies conducted in diverse countries such as Saudi Arabia (105), Bangladesh (27, 29), Colombia (58), Thailand (32, 34), and Malaysia (106). However, it contrasts with the findings of Suwanbamrung et al. (48), which showed that teachers were the main source of information on this topic. This reliance on traditional channels presents both a challenge and an opportunity: public health campaigns must leverage digital platforms to reach broader and younger audiences, thereby enhancing the effectiveness and scalability of MBD prevention efforts.

This study highlights a critical gap between general awareness of MBD and specific biomedical knowledge, with only 21.5% of participants correctly identifying dengue as a viral disease despite widespread recognition of mosquito transmission. This disconnect mirrors global trends and underscores the urgent need for targeted educational strategies that move beyond symptom recognition to foster pathogen specific understanding, which is essential for effective prevention and timely healthcare-seeking behavior (32, 58).

A majority of respondents recognized the importance of seeking medical attention for the diagnosis of MBD, whether at hospitals, private physicians’ offices, or public health centers. This finding is consistent with research conducted in Cameroon (104) and Colombia (70). In contrast, studies in Mexico have found that traditional medicine and home remedies are often preferred (103). The high level of knowledge about mosquito life cycles (91.9%) stands in stark contrast to the low recognition of clinical treatment protocols. This pattern is similar to results observed in Tanzania and Bangladesh, where ecological knowledge tends to surpass biomedical knowledge (29, 49). Notably, the reliance on television (31.4%) and interpersonal networks (a combined 43.6%) as primary sources of information highlights the continued influence of traditional communication channels, as opposed to the widespread use of digital platforms observed elsewhere (39, 44).

The most commonly reported preventive measure was the elimination of stagnant water, which is consistent with findings from other studies such as Alghazali et al. (107) and Desjardins et al. (57). However, some studies have found a preference for mosquito nets (18, 29, 50, 105) or chemical based methods like insecticides (104, 108). The strong emphasis on environmental management evidenced by 84.9% of participants prioritizing the elimination of stagnant water indicates effective public health messaging. Nevertheless, this contrasts with lower engagement in personal protection measures, a pattern also observed in urban communities in Brazil and Mexico (13, 51). Notably, only 58.7% of respondents acknowledged the existence of treatments for mosquito-borne diseases, suggesting potential gaps in healthcare access or distrust in medical systems a phenomenon previously associated with marginalized populations in Peru and Kenya (50, 56).

Regarding responsibility for MBD prevention, most participants believed it should be an individual responsibility, followed by actions carried out by the municipal administration (47). In this study, responses demonstrated high levels of knowledge and good practices in the management of MBD, similar to findings reported by Cochero et al. (59), Udayanga et al. (67), Phuyal et al. (68), and Barua et al. (69). A striking finding is the pronounced disconnect between high levels of knowledge and the prevalence of indifferent attitudes (94.8%), which fails to translate into consistent preventive practices (70). This gap signals that informational interventions alone are insufficient; psychosocial barriers, low risk perception, and limited institutional trust must be addressed through multifaceted strategies that integrate community empowerment and robust public sector engagement (66, 68).

Despite high literacy rates, the prevalence of indifferent attitudes (94.8%) challenges the assumption of a direct link between knowledge and behavior. This finding supports the hypothesis of van den Berg et al. (24) that psychosocial and infrastructural factors mediate the relationships between KAP. Indifference toward MBD and mosquitoes should be critically examined, considering possible underlying factors such as lack of knowledge, low risk perception, information fatigue, and cultural or socioeconomic barriers. Public health policy must prioritize interventions that bridge the gap between knowledge and action. This requires not only improving biomedical literacy but also fostering community engagement, building institutional trust, and deploying digital tools for health communication. Policies should be informed by local KAP profiles and designed to empower communities as active partners in disease prevention, ensuring that individual efforts are matched by effective organizational support.

This research provides evidence supporting the hypothesis that urban populations in MBD endemic regions develop selective health literacies shaped by cultural narratives and historical exposure to outbreaks. Our principal contribution is quantifying the gap between knowledge of mosquito control (91.9%) and pathogen specific understanding (21.5%), which represents a critical barrier to effective clinical prevention. The finding that 51.7% of participants desire more information about MBD offers an actionable pathway for community engaged interventions, particularly through mobile platforms that are already widely adopted.

The multinomial logistic regression model revealed a significant association between higher knowledge levels and participants’ marital status, in line with findings by Selvarajoo et al. (38) and Naing et al. (71), which demonstrated that being married is often linked to greater knowledge of mosquito-borne disease (MBD) management. In this study, both unmarried, married, and cohabiting individuals exhibited high knowledge, potentially due to differing life circumstances: unmarried individuals, typically younger, may access more recent information through academic environments, while married or cohabiting adults are more likely to participate in environmental education workshops as part of their employment (38). Furthermore, marital or partnered relationships can facilitate a positive feedback loop and mutual transfer of knowledge, especially when children are present, heightening concern for family health and well-being (72–75). However, despite these associations with knowledge, neither age nor marital status predicted attitudes or practices, underscoring that knowledge alone does not necessarily translate into behavior change. Consequently, effective interventions must go beyond demographic targeting to address broader structural and psychosocial determinants that shape health-related behaviors at the community level.

While the KAP framework has proven valuable for understanding community responses to mosquito-borne diseases, contemporary public health discourse increasingly situates such assessments within broader ecological paradigms. The concept of Nature Quotient (NQ), which emphasizes humanity’s capacity to coexist harmoniously with nature (76), offers a complementary lens through which to interpret our findings. From this perspective, cultivating public KAP regarding MBD represents a subdomain within the larger endeavor of nurturing societal ecological intelligence and fostering sustainable coexistence with natural ecosystems (61, 76). This ecological intelligence manifests not only in everyday behaviors and perceptions but also in transformative public health achievements, exemplified historically by Tu Youyou’s discovery of artemisinin from traditional herbal knowledge (76). Our findings reveal that participants possessed substantial knowledge about mosquito ecology (91.9%) yet demonstrated limited understanding of pathogen-specific mechanisms (21.5%), suggesting that ecological literacy alone is insufficient without deeper biomedical integration. To bridge this gap and enhance NQ at the community level, targeted education and communication strategies become paramount (68, 77, 78).

Evidence from systematic reviews and field interventions demonstrates that effective health education for MBD prevention requires multifaceted, culturally resonant approaches that transcend traditional information dissemination (68, 78, 79). Community-based participatory education, involving workshops, peer educators, and household visits, has consistently demonstrated efficacy in improving knowledge scores, reducing vector indices, and promoting sustained preventive behaviors across diverse settings (79–82). School-based health promotion programs utilizing interactive methodologies and audiovisual materials have proven particularly effective, as students serve as change agents who amplify health messages throughout their families and communities (14, 83–85). Innovative communication strategies, including narrative storytelling and digital health technologies, show promise for engaging underserved populations and overcoming psychosocial barriers to behavior change (68, 86–88). Storytelling approaches that incorporate culturally specific narratives, personal testimonies, and traditional communication mediums resonate more deeply with communities, fostering emotional engagement and facilitating the translation of knowledge into practice (68, 86, 89). Digital platforms, including mobile health applications, SMS-based interventions, and participatory surveillance tools, offer scalable solutions for real-time disease monitoring, rapid information dissemination, and bidirectional communication between health authorities and communities (88, 90–92).

Integrating these educational and communicative strategies within the One Health framework enhances their effectiveness by addressing the multidimensional drivers of vector-borne diseases (77, 93–96). The One Health approach recognizes that human health, animal health, and environmental health are interconnected and that collaborative, multisectoral interventions yield superior outcomes (93–98). Successful implementation requires strong governance, sustained funding, community empowerment, and the strategic use of digital technologies for integrated surveillance and response (77, 90, 95, 98, 99). In our study context, the pronounced disconnect between high knowledge levels and prevalent indifferent attitudes (94.8%) underscores the necessity of moving beyond cognitive interventions to address affective and contextual determinants of health behavior. Future interventions should prioritize participatory approaches that co-design solutions with communities, leverage culturally appropriate communication channels, integrate digital tools for sustained engagement, and embed MBD prevention within broader environmental health and sustainability education (78–80, 97, 100, 101). By reframing MBD prevention as an integral component of developing societal NQ, public health programs can foster ecological consciousness that supports not only disease control but also broader goals of environmental stewardship and One Health implementation (76, 77, 94, 96).

Finally, it is important to highlight Colombia’s commitment to addressing public health challenges through community based and empirical research. Strengths of this study include the use of information and communications technologies and the review of novel documentation on MBD as potential keys for future health management interventions. A key limitation of this study is its cross-sectional design and urban, highly educated sample, which restricts the generalizability of findings to rural or less-educated populations where the burden of MBD may be greater (11). Additionally, while the sample size was sufficient for estimating population proportions, it may have limited the statistical power and generalizability of our multinomial logistic regression analyses, particularly in subgroup comparisons. Future research should employ larger, stratified samples along with mixed methods and validated, disease-specific instruments to disentangle the drivers of attitudinal indifference and assess the long-term impact of digital and community-based interventions.

The KAP strategy has been successfully implemented in a number of tropical regions around the world. The results obtained in these regions are consistent with those from local communities in other countries, including significant studies from the Lao Democratic Republic, Thailand (34) and Hong Kong (37). In Colombia, KAP surveys have been instrumental in exploring the interaction between sociodemographic factors and community health behaviors, particularly in urban centers such as Riohacha (56) and Cali (57, 66). The data processing methods used, particularly multivariate analysis, enhance the robustness of the results and contribute to the refinement of MBD prevention strategies and mosquito management practices. This analytical approach not only facilitates a deeper understanding of community dynamics but also informs targeted interventions that are critical for effective disease control.

The existence of unanswered questions highlights the need for further research. First, how do socioeconomic gradients within urban areas influence KAP profiles? Second, it is important to determine the role of misinformation in sustaining indifferent attitudes despite high literacy rates. Third, could the strong emphasis on individual responsibility (90%) potentially hinder collective action against mosquito proliferation? Future studies should employ mixed methods to explore cultural perceptions of MBD and assess the long-term effectiveness of digital education campaigns in bridging the KAP gap.

## 5 Conclusion

The findings indicate that while marital status is significantly associated with knowledge levels and education shows a positive trend, no sociodemographic factors influence attitudes or the adoption of preventive practices against mosquito-borne diseases. There is a general awareness of basic control measures but limited understanding of the viral etiology and a prevailing indifference that fails to translate into personal protection. This underscores that mere information dissemination is insufficient to effect behavioral change; therefore, context-specific educational interventions are needed to foster active engagement and institutional trust, address psychosocial and risk-perception barriers, and leverage digital tools and school settings to empower communities and bridge the gap between knowledge and practice.

## Data Availability

The raw data supporting the conclusions of this article will be made available by the authors, without undue reservation.

## Footnotes

^1^ https://es.surveymonkey.com/mp/sample-size-calculator/

^2^ https://forms.gle/bCgM1o2ZZAW5GmsMA
