# Supplementary material for: Knowledge, attitudes, and practices regarding mosquito-borne diseases in an urban sector of southwestern Colombia
Source: Front Public Health. 2025 Dec 3;13:1682827. doi: 10.3389/fpubh.2025.1682827 (PMC12708523; doi:10.3389/fpubh.2025.1682827)
Supplement: Supplementary file 1 [file Table_1.docx]

Supplementary Material

# Supplementary Tables

**Supplementary Table 1.** Knowledge, Attitudes and Practices (KAP) survey on mosquito-borne diseases, adapted from PAHO (2016).

| Informed consent | |
| --- | --- |
| I, as an adult, agree to participate voluntarily and anonymously in a ‘survey’ that aims to understand the knowledge, attitudes and practices (KAP) that the urban community has about mosquito control, what they think about it and how they apply prevention.  I declare that I have been informed of the objectives and procedures of the study and of the type of participation that I will be asked to provide: answers about myself, my environment, school, family, friends, with the aim of strengthening the skills of the communities on these issues.  I authorize to participate in the following procedures:  a. Collection of relevant data on their general development, academic history, family history or other data that may be important.  b. In general, to allow the collection of information through filming, photographic recordings, questionnaires, among others. I declare that I have been informed that participation in this study does not involve any harm or danger to physical or mental health, that it is voluntary and that I can refuse and stop participating at any time without explanation or penalty.  Furthermore, I declare that I will not benefit from participating in this study, however, the information that may be obtained from my participation will be useful for the prevention of mosquito-borne diseases (dengue, Zika, chikungunya, malaria) in the community. Participating in this study will also involve no expenses for me and I will not receive any payment for being in this study.  I declare to know that the information provided will be confidential and anonymous, there will be no way to identify the data. All information obtained and results will be treated confidentially.  I hereby declare that this document has been read and understood by me in its entirety in a free and spontaneous manner.   - If I authorize ________. | |
| **Socio-demographic characteristics of participants** | |
| 1. Gender   - Woman - Man - I prefer not to say - Other:   2. Age   - 18 to 27 years old - 28 to 37 years old - 38 to 47 years old - 48 years and over   3. Marital Status   - Unmarried - Married - Free union - Separated - Widowed   4. Level of education   - Primary school - Secondary - Technician - Technologist - University - Other   5. Occupation   - Employed - Self-employed - Various trades - Other | 6. Number of dependents   - None - 1 to 3 persons - 4 to 6 persons - More than 6 persons   7. Average monthly income   - None - Legal minimum wage (L.M.W) - Between 1 to 2 L.M.W. - Between 3 to 4 L.M.W. - More than 7 L.M.W.   8. Number of women of childbearing age (women aged 15-49 years) in the household   - None - 1 - 2 - 3   9. Number of pregnant women in the household   - None - 1 - 2 - 3 |
| **Knowledge of mosquito-borne diseases (MBD)** | |
| 1. Are you familiar with the term "mosquito-borne disease" (MBD)?    - Yes / No 2. Do you understand the life cycle of mosquitoes?    - Yes / No 3. When did you first learn about mosquito-borne diseases?    - More than a year ago / Within the last year / Within the last few months / Within the last month / I don't recall 4. From which of the following sources did you FIRST learn about mosquito-borne diseases? (Select ONE)    - Family/Friends / Healthcare provider / Media (TV, Radio, Internet) / Public health campaign / Other / Don't recall 5. How likely do you think it is to contract a mosquito-borne disease in your community?    - Very likely / Likely / Indifferent / Unlikely / Very unlikely / Don't know 6. Have you personally known anyone who has contracted a mosquito-borne disease in your community in the past year?    - Yes / No / Don't know | 1. Who is most at risk of contracting a mosquito-borne disease? (Select all that apply)    - Children / Pregnant women / Elderly individuals / People with weakened immune systems / Everyone is equally at risk / Don't know 2. Dengue fever is primarily transmitted through:    - Mosquito bites / Contaminated food or water / Direct contact with an infected person / Don't know 3. Mosquito-borne diseases are MOSTLY transmitted by:    - Infected insect bites / Contaminated food or water / Air droplets / Don't know 4. Which of the following are common symptoms of dengue fever? (Select all that apply)    - High fever / Severe headache / Joint and muscle pain / Skin rash / Don't know 5. Which of the following are effective ways to prevent mosquito-borne diseases? (Select all that apply)    - Using mosquito repellent / Wearing long sleeves and pants / Eliminating standing water / Using mosquito nets / Don't know 6. Is there a specific cure for most mosquito-borne viral infections?    - Yes / No / Unsure |
| **Attitudes on mosquito-borne diseases (MBD)** | |
| 1. Mosquito-borne diseases are a significant problem in my community.    - Strongly Disagree / Disagree / Indifferent / Agree / Strongly Agree 2. I am concerned about my risk of contracting a mosquito-borne disease in the next 6 months.    - Strongly Disagree / Disagree / Indifferent / Agree / Strongly Agree 3. I would prefer to keep it confidential if a family member contracted a mosquito-borne disease.    - Strongly Disagree / Disagree / Indifferent / Agree / Strongly Agree 4. People who contract mosquito-borne diseases and their families may face discrimination in my community.    - Strongly Disagree / Disagree / Indifferent / Agree / Strongly Agree 5. Who do you believe is MOST responsible for preventing mosquito-borne diseases in your community? (Select ONE)    - Individuals/Households / Community Leaders / Healthcare Providers / Local Government / National Government / Don't know | 1. I believe that a pharmacy can effectively treat mosquito-borne diseases.    - Strongly Disagree / Disagree / Indifferent / Agree / Strongly Agree 2. I believe that a public health center can effectively treat mosquito-borne diseases.    - Strongly Disagree / Disagree / Indifferent / Agree / Strongly Agree 3. I believe that a public hospital can effectively treat mosquito-borne diseases.    - Strongly Disagree / Disagree / Indifferent / Agree / Strongly Agree 4. I believe that a local healer can effectively treat mosquito-borne diseases.    - Strongly Disagree / Disagree / Indifferent / Agree / Strongly Agree 5. I believe that a private doctor can effectively treat mosquito-borne diseases.    - Strongly Disagree / Disagree / Indifferent / Agree / Strongly Agree 6. I feel I am well-informed about mosquito -borne diseases.    - Strongly Disagree / Disagree / Indifferent / Agree / Strongly Agree |
| **Practices on mosquito-borne diseases (MBD)** | |
| 1. In the past year, have you or your family taken any specific actions to prevent mosquito-borne diseases?    - Yes / No / Don't know 2. Which of the following actions have you taken to protect yourself and your family from mosquito-borne diseases in the past year? (Select all that apply)    - Used mosquito repellent / Worn protective clothing (long sleeves, pants) / Eliminated standing water around the home / Used mosquito nets / Other / None 3. I am aware of mosquito -borne disease prevention efforts by my local municipal administration.    - Yes / No / Unsure 4. I have seen or heard about government programs to prevent mosquito-borne diseases in my community.    - Yes / No / Unsure | 1. I have seen or heard about programs from local or national organizations to prevent mosquito-borne diseases in my community.    - Yes / No / Unsure 2. Which of the following methods can help reduce or eliminate mosquitoes around your home? (Select all that apply)    - Using insecticide sprays / Removing standing water / Maintaining clean surroundings / Using mosquito traps / Don't know 3. How often do you clean or empty water storage containers around your home?    - Weekly / Monthly / Less often than monthly / Never 4. If a safe and effective vaccine were available for common mosquito-borne diseases, I would get vaccinated.    - Strongly Disagree / Disagree / Indifferent / Agree / Strongly Agree |
